# Convergence of physiological responses to pain during face-to-face interaction

**DOI:** 10.1038/s41598-019-57375-x

**Published:** 2020-01-16

**Authors:** Aiko Murata, Hiroshi Nishida, Katsumi Watanabe, Tatsuya Kameda

**Affiliations:** 1 NTT Communication Science Laboratories, NTT Corporation, Atsugi, Japan; 2 Department of Behavioral Science, Hokkaido University, Sapporo, Japan; 3 Faculty of Arts and Science, Kyushu University, Fukuoka, Japan; 4 Faculty of Science and Engineering, Waseda University, Tokyo, Japan; 5 Art and Design, University of New South Wales, Sydney, Australia; 6 Faculty of Letters, the University of Tokyo, Tokyo, Japan; 7 Center for Experimental Research in Social Sciences, Hokkaido University, Sapporo, Japan; 8 Brain Science Institute, Tamagawa University, Machida, Japan

**Keywords:** Cardiovascular biology, Human behaviour

## Abstract

Empathy with another’s pain is an important social glue for maintaining interpersonal relationships. In most previous studies investigating the sharing of pain, a signal conveying a painful experience is presented by a target (“sender”) as a stimulus to a participant (“receiver”), and the emotional/physiological responses of the participant are measured. However, this unilateral “sender-receiver” paradigm does not adequately address the possible bidirectional experience of shared pain accruing from interaction. Our aim was therefore to investigate the bidirectional effects of sharing pain in social settings. Thirty-six unfamiliar pairs were simultaneously and repeatedly exposed to the same pain-provoking (thermal) stimuli, either in a face-to-face or a “shielded” condition where a partition prevented the partner’s responses from being fully observed. We recorded the blood volume pulse of each participant to measure the acute sympathetic response while a pair of participants experienced the stimuli simultaneously. The results revealed that participants with weaker reactions elevated their physiological reactivity to the stimulus in accordance with their partner’s reactions in the face-to-face condition. The pair-level physiological similarity was also higher compared to the shielded condition. Such a low-to-high physiological convergence may underlie the collective elevation of pain expressions, which is often observed in interactive settings.

## Introduction

Pain is a sensory and emotional experience associated with actual or potential tissue damage^1^. It is thus often seen as personal, subjectively felt experience. However, neuroscience research has shown that observing someone in a painful situation activates neural responses related to the observer’s own experience of pain. For example, when an individual observes the application of electric stimulation to a partner’s hand, neural regions associated with her/his own pain (e.g., anterior insula, anterior cingulate cortex) are activated in the observer^2,3^. Such vicarious responding to another’s experiences is considered to be one of the core building blocks of social relations^4,5^. Most previous studies of the psychological and neural mechanisms behind the sharing of pain have used an experimental paradigm in which a signal conveying a painful experience is presented by a target (“sender”) as a stimulus to a participant (“receiver”), and the emotional/physiological responses of the participant were measured^2,3,6–9^. However, in actual social interaction, individuals do not only “receive” other’s emotions, but also “send” their own. Emotional responses that are evoked by the observation of another’s emotional expression (e.g., facial expression, body movement), could also influence the other’s emotional state in a bilateral manner. Such psychophysiological dynamics emerging from social interaction^10,11^ cannot be captured by the unilateral “sender-receiver” paradigm.

To our knowledge, there have been few human studies that address the bidirectional dynamics of shared pain. For non-human animals, Langford *et al*.^12^ showed that mice could share pain with their partners. In their experiment, painful stimuli were applied to two mice simultaneously, and their behavioural responses (e.g., writhing, licking) were measured. They found that the mice writhed more when they received a painful stimulus simultaneously with the other mouse, compared to when receiving it alone. The mice were especially influenced when paired with a familiar cage mate, and their own pain reactivity increased, which suggests a bidirectional amplification of pain. This effect was weaker when a mouse was paired with an unfamiliar other, and even disappeared when the mice were visually impaired and thus unable to see the other mouse.

Martin *et al*.^13^ extended this paradigm to humans, demonstrating that participants reported greater subjective pain when they experienced the pain together, compared with experiencing it alone. Thus, humans also seem to show a similar effect to that observed in mice by Langford *et al*.^12^. Furthermore, in line with Langford *et al*.^12^, the effect disappeared when the pairs consisted of strangers. Interestingly, when a drug was used to reduce the social stress caused by interacting with an unfamiliar person, the stranger pairs also reported greater subjective pain compared to the solo participants.

Although these studies are important for understanding the bidirectional aspect of pain experience at a behavioural or subjective level, the way in which interpersonal influence may occur at the physiological level remains unclear. According to the James–Lange-type theories of emotion^14,15^, physical responses are evoked prior to subjective evaluations of feelings. To verify whether the sharing of pain occurs not only at the behavioural level (e.g., convergence of emotional expressions) but also operates at deeper levels, it is essential to measure the physiological aspects of pain (which are difficult to consciously control) during a bilateral interaction. For this purpose, we recorded the blood volume pulse (BVP) using a finger photoplethysmogram of each participant in a pair while they simultaneously experienced pain-provoking thermal stimuli. BVP has been used to assess rapid stimuli–induced autonomic responses^16^, and is suitable for capturing an individual’s acute physiological response to a pain-provoking stimulus. Specifically, a reduction in BVP amplitude from the baseline in response to a stimulus reflects peripheral vasoconstriction in the finger and is known to be associated with sympathetic arousal caused by acute stress^16,17^ and thermal pain^18^. We thus used the constriction percentages of BVP amplitude after stimulus onset as an index of physiological pain response (see Methods for details).

**Figure 1:**
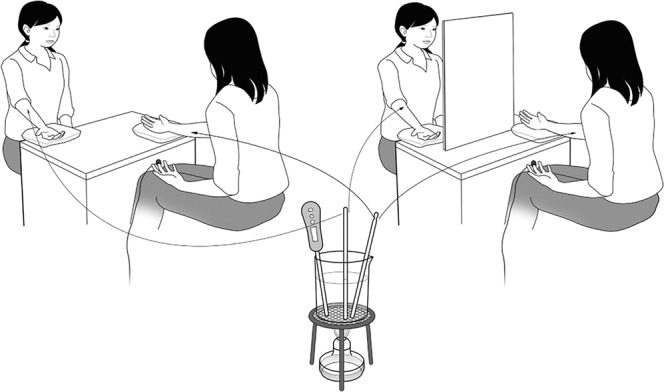
Experimental Setup. In the face-to-face condition, the two participants were seated facing each other. They could view their partner’s responses (left picture). In the shielded condition, a small partition was placed between them (right picture), which blocked any view of the partner’s upper body (including the face) but did not completely block the view of the partner’s hand. The participants were instructed to direct their gazes forward during the task under both conditions, so they could see their partner’s hand in their peripheral visual field. Aluminium rods were heated in hot water using a spirit lamp and applied simultaneously to the right arm of each participant for about 300 milliseconds. Because we heated the two stimuli in the same transparent beaker, the participants knew they experienced the stimulus at the same temperature as their partner.

There are three possibilities regarding the way physiological convergence may occur in pairs: (1) the weaker-responding person in the pair elevates their responses, (2) the stronger-responding person lowers their responses, (3) both people adjust their responses toward each other. Given the previous findings that, on average, greater pain response was exhibited in a pair situation compared to a solo situation^12,13^, we conjecture that the low-to-high convergence, (1) above, is most likely. To test these possibilities, we measured the physiological response when paired participants were simultaneously and repeatedly exposed to the same series of pain-provoking thermal stimuli. The thermal stimuli, which were increased from moderate to higher temperatures in 5 degree steps (40 °C, 45 °C, 50 °C, 55 °C, 60 °C, 65 °C), were applied sequentially to the participant’s right arm a total of 12 times, twice for each level (see Fig. 1). We assessed the convergence within a pair in two ways: (a) inter-trial influence where one’s physiological response to the current stimulus may be influenced by the partner’s response to the previous stimulus in the sequence, and (b) intra-trial similarity where physiological responses to the same stimulus may be correlated between the two participants.

To examine the effect of real-time interaction on physiological convergence, we adopted two between-participant conditions: a face-to-face condition where each participant could see their partner’s responses, and a shielded condition where a small partition prevented the participants from fully viewing their partner’s responses (Fig. 1). In the shielded condition, the participants knew that they were experiencing an applied stimulus of the same temperature as their partner’s simultaneously by observing the experimenter’s actions and their partner’s hand. The participants were not allowed to communicate verbally during the task in either condition. Thus, the difference between the face-to-face and shielded conditions was mainly whether or not the other’s facial expressions (e.g., eye movement, facial muscular activity) were visible. To control the familiarity of the pairs, all the participants were recruited individually so that they had no interactions prior to the experiment (i.e., unfamiliar pairs), and were paired with someone of the same sex. The participants were given an opportunity for a short conversation before the task to reduce social stress that might arise from interacting with an unfamiliar person^13^.

## Results

### Inter-trial physiological influence in pairs

As an index of acute physiological responses to the pain-provoking thermal stimulus, the constriction percentages of BVP amplitude after the stimulus onset were analysed with a linear mixed-effects model (LMM) using the “lmer” function in the “lme4” package for R^19^. In this analysis, we examined the influence of the partner’s BVP response to the previous stimulus (e.g., 40 °C, 45 °C, 50 °C, 55 °C, 60 °C) on the participant’s BVP response to the current stimulus (e.g., 45 °C, 50 °C, 55 °C, 60 °C, 65 °C) in the sequence (e.g., whether the participant’s response to 50 °C was influenced by the partner’s response to 45 °C). To identify the direction of social influence (i.e., low-to-high convergence, high-to-low convergence, or symmetric), we defined participant’s “relative position” (i.e., the weaker or the stronger responder) according to her/his BVP response to the previous stimulation. If the influence is symmetric, the participant’s BVP response to the current stimulus should be predicted by the partner’s BVP response to the previous stimulus equally regardless of relative position within a pair. On the other hand, if low-to-high convergence characterises social influence, the weaker responder’s current BVP response should be elevated by the previous response of the partner (who exhibited the stronger response in the previous stimulation), but not vice versa.

In the LMM for participant’s BVP response, the condition (face-to-face or shielded), participant’s relative position (the weaker or the stronger responder), stimulus (45 °C, 50 °C, 55 °C, 60 °C, 65 °C) and the partner’s BVP response to the previous stimulus, and their interactions were entered as fixed effects. Participant and pair were entered as random effects.

A Wald Chi-Square test using the LMM revealed the following effects. Most important to our interest, social influence was moderated by both condition and participant’s relative position – the Condition × participant’s relative position × partner’s previous response interaction was significant: *χ*2(1) = 4.545, *p* = 0.033. (Two other effects were also significant: condition × stimulus, *χ*2(1) = 4.622, *p* = 0.032, and a main effect of participant’s relative position, *χ*2(1) = 7.616, *p* = 0.006, implying that the weaker responder in the previous stimulus yielded weak responses to the current stimulus as well. The parameter coefficients of the model are shown in Supplementary Table S1.)

The three-way interaction means that social influence between the weaker and the stronger responder in a pair was not symmetric (i.e., moderated by the participant’s relative position) and that this asymmetric pattern was further moderated by condition (face-to-face or shielded). To see the interaction effect in detail, below we report separate post-hoc LMM analyses for each condition.

### LMM for face-to-face condition

**Figure 2:**
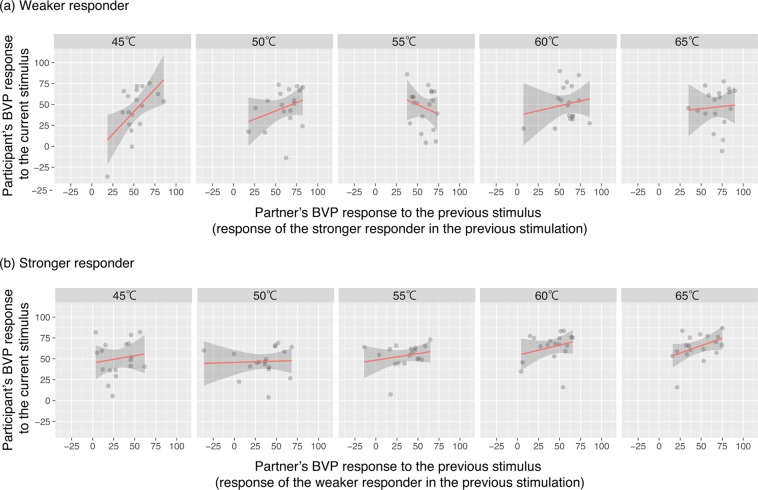
Influence of partner’s BVP response on the participant’s response in a face-to-face condition, when (**a**) the participant was the weaker responder or (**b**) the stronger responder in the pair. The *grey points* represent scatterplots showing the relationship between a participant’s BVP responses to the current stimulus and their partner’s responses to the previous stimulus. The *red lines* and *shaded areas* represent linear predictions and 95% confidence intervals.

To examine how asymmetric influence operated in the face-to-face condition, we plotted the participant’s BVP responses to the current stimulus against her/his partner’s BVP response to the previous stimulus, when the participant was the weaker responder (Fig. 2a) or the stronger responder (Fig. 2b). As seen in the figure, the slopes of early stimulations in the sequence (e.g., 45 °C, 50 °C) indicated a pattern corresponding to the low-to-high convergence: the current BVP responses of the weaker responders were positively correlated with their stronger partners’ responses to the previous stimulus (Fig. 2a), but not vice versa (Fig. 2b).

The LMM analysis supported this observation. A Wald Chi-Square test revealed a significant main effect of the stimulus (*χ*2(1) = 11.941, *p* = 0.001, Bonferroni adjusted), the partner’s response × participant’s relative position interaction (*χ*2(1) = 7.645, *p* = 0.011, Bonferroni adjusted), and the stimulus × the partner’s response × participant’s relative position interaction (*χ*2(1) = 8.797, *p* = 0.006, Bonferroni adjusted). Notice that the main effect for the partner’s response was not significant (*χ*2(1) = 1.583, *p* = 0.416, Bonferroni adjusted) while the partner’s response × participant’s relative position interaction was significant. This means that social influence was asymmetric: as seen in the figure, the weaker responders elevated their BVP response in accordance with the partner’s (i.e., the stronger responder’s) previous response, but not vice versa. Moreover, this low-to-high convergence weakened in later stimulations in the sequence, as indicated by the three-way interaction (see Supplementary Table S2 for parameter coefficients of the model).

### LMM for shielded condition

A Wald Chi-Square test using the LMM for the shielded condition revealed no significant effects except for a main effect of participant’s relative position (*χ*2(1) = 6.964, *p* = 0.008). This effect simply means that the weaker responder in the previous stimulus was likely to show a weaker response to the current stimulus as well (parameter estimates are shown in Supplementary Table S3). Unlike the face-to-face condition, no physiological influences of partner’s responses were observed in the shielded condition.

Taken together, these results indicate that a physiological influence occurred in the face-to-face interaction, such that the weaker responders elevated their BVP responses according to their partner’s response to the previous stimulus. Furthermore, this low-to-high convergence weakened in later stimulations in the sequence, indicating that as the weaker participant’s responses caught up with the higher responses in the pair, the physiological influence gradually diminished. Moreover, this asymmetric convergence from low to high appears to have been facilitated by viewing the other’s facial expressions, which was only possible in the face-to-face condition.

### Intra-trial physiological similarity in pairs

In addition to the inter-trial social influence, we examined intra-trial similarity in physiological changes (i.e., BVP waves) between paired participants. To determine whether BVP waves of paired participants become similar as a result of social interaction, it is necessary to separate the similarity caused by interaction from that caused by the common thermal stimulus alone. Therefore, we compared the BVP wave similarities of actual pairs with those of nominal pairs consisting of two participants under the same condition but in different sessions. Since the members of a nominal pair received the same stimulus but did not interact during the task, any BVP wave similarity arose purely from the stimulus alone; additional similarities between actual pairs compared with nominal pairs can thus be attributed to interaction.

BVP waves of paired participants during a 19-second period covering before and after stimulus onset (examples of BVP waves are shown in Methods) were used for calculating the BVP wave similarity in each of the 12 trials. Because the sampling rate of the BVP wave was 100 Hz, there were 1900 data points per trial. We conducted a cross correlation analysis for each trial in each pair, which measured the similarity of two shifted discrete signals as a function of the time lag. Since spontaneous emotional contagion is known to be evoked within 1000 ms of observing an emotional stimulus^20–23^, we adopted a peak value for the correlation coefficients of a −1000 millisecond lag to a + 1000 millisecond lag as an index of BVP wave similarity for each pair.

**Figure 3:**
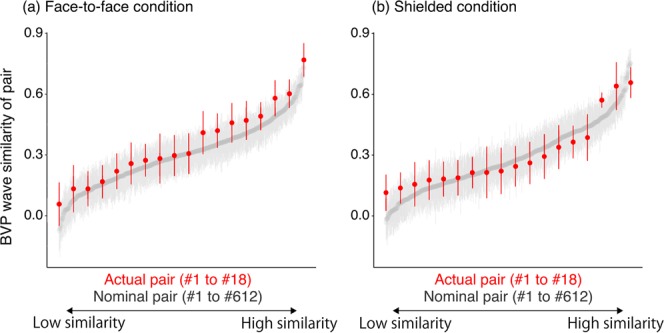
BVP wave similarities in actual pairs and nominal pairs: (**a**) face-to-face condition, and (**b**) shielded condition. The *red points* represent the average BVP wave similarity (the peak value of the correlation coefficients from a −1000 millisecond lag to a +1000 millisecond lag) of each of the 18 actual pairs. The *grey points* represent the average BVP wave similarity of each of the 612 nominal pairs. The pairs were ordered from low to high in terms of the mean value of the BVP wave similarity. The nominal pairs are all of the possible combinations of two participants who took part in different sessions under the same condition (_36_C_2_ −18 = 612 pairs under each condition). The error bars indicate standard errors.

Figure 3 shows the mean BVP wave similarities of each actual pair and nominal pair under the two conditions. The pairs were ordered from low to high in terms of the mean value of the BVP wave similarities. Under the face-to-face condition (Fig. 3a), actual pairs (red points) showed a higher similarity than nominal pairs (grey points). This observation was confirmed by one-sided Kolmogorov-Smirnov tests (*D* = 0.102, *p* = 0.013). Under the shielded condition (Fig. 3b), however, there was no significant difference between the actual and nominal pairs (*D* = 0.049, *p* = 0.371). Furthermore, the actual pairs showed a greater similarity under the face-to-face condition than under the shielded condition (*D* = 0.125, *p* = 0.034). These results clearly indicate that face-to-face interaction yielded an intra-trial physiological similarity above and beyond the similarity originating from the common thermal stimulus.

### Physiological similarity and personality traits

Thus far, we have reported significant differences from group level analysis by comparing face-to-face and shielded conditions. However, it should be noted that the degree of physiological similarity varied substantially across the pairs even under the face-to-face condition (Fig. 3a). Such differences in the pair-level physiological similarities may be due to individual trait differences composing each pair. For example, at the individual level, a relationship between empathic personality traits and unilateral empathic response has been observed in previous research^2,6,24^. Here, we explored the relevance of autism-spectrum quotient (AQ^25)^ scores and trait empathy (Interpersonal Reactivity Index: IRI^26)^. To shed light on how individual trait differences may be mapped onto the pair-level phenomenon, we examined whether the higher personality score in a pair (e.g., the maximum), the lower personality score in a pair (the minimum) or both may affect the physiological similarity at the pair level (i.e., the BVP wave similarities reported in Fig. 3).

*AQ*. To examine whether lower AQ (higher social sensitivity), higher AQ (lower social sensitivity), or both influence the degree of overall physiological similarity, we conducted two LMM analyses. In each model, the minimum or maximum score in each pair and the condition were entered as fixed effects. Pair and trial were entered as random effects. If the AQ scores of paired participants affect physiological similarity during face-to-face interaction, the effect of AQ will be observed under the face-to-face condition, but not under the shielded condition.

*Model with minimum AQ in pair*: a Wald Chi-Square test for the model with the minimum AQ score revealed a significant interaction between condition and AQ (*χ*2(1) = 6.104, *p* = 0.027, Bonferroni adjusted), while the main effects of AQ (*χ*2(1) = 0.629, *p = *0.856, Bonferroni adjusted) and condition (*χ*2(1) = 0.092, *p* = 1.000, Bonferroni adjusted) were not significant. In particular, the lower the pair’s minimum AQ, the higher the physiological similarity of the pair became under the face-to-face condition (β = −0.022, *95% CI:* −0.041 to −0.004). By contrast, we found no clear effect of the minimum AQ on the physiological similarity under the shielded condition (β = 0.006, *95% CI:* −0.008 to 0.018).

*Model with maximum AQ in a pair*: we found no significant effects in the model with the maximum AQ score (AQ: *χ*2(1) = 1.313, *p* = 0.504, condition: *χ*2(1) = 3.553, *p* = 0.119, the interaction: *χ*2(1) = 4.061, *p* = 0.088, Bonferroni adjusted, respectively).

These results indicate that the social sensitivity (measured by AQ) of paired participants is related to the physiological similarity of the pair, which may imply that those who are more socially sensitive (lower AQ) pay closer attention to their counterpart’s responses, thus increasing the physiological similarity of the pair.

*Trait empathy*. Empathic traits were also examined with LMMs. However, no effects reached statistical significance (all *p* > 0.05).

## Discussion

In everyday life, we experience emotions not only individually but also interactively. However, with a few exceptions^12,13^, most previous research on pain-sharing has centred on unilateral situations by employing the sender-receiver paradigm. In this study, we showed that the sharing of pain occurred at the physiological level during face-to-face, bilateral interaction. The face-to-face interaction yielded social influence in which the weaker responder elevated their physiological (BVP) responses according to their partner’s response to the previous stimulus. In addition to such inter-trial “low-to-high” convergence, we found intra-trial similarity where the physiological signals of the paired participants in each trial were similar above and beyond the similarity caused by the shared stimulus per se. In contrast, we observed no such effects under the shielded condition. These observations suggest that a face-to-face situation is critical for the sharing of pain at the physiological level.

Martin *et al*. have demonstrated that pairs of people experiencing painful stimuli together report a greater degree of subjective pain than solo participants^13^. The low-to-high physiological convergence we found here may be one likely dynamic underlying such a pain-amplification effect through interaction. When two people have different sensitivities to pain, a convergence in which the two approach each other symmetrically would not yield an amplification effect, whereas low-to-high asymmetric convergence would. We also speculate that such low-to-high convergence may have some adaptive basis. In a natural environment, where failing to notice a critical change or threat often has more harmful consequences than a false alarm, the emotional expression of a conspecific who shares the same environment can provide valuable information for detecting and preparing for a change^27,28^. In such cases, adjusting one’s physical state to the stronger emotional expressions of others would be ecologically rational.

It is also important to note that social settings can differ in terms of the interactions involved. In most previous studies that used the sender-receiver paradigm^2,3,6–9^, no interaction was allowed, so that only the receiver could be influenced by the pain expressions of the sender. In many situations, however, the receiver may be allowed to express compassion or empathic concern to the sufferer. For example, previous neurophysiological studies showed that, a person who held another’s hand while receiving a painful stimulus was able to regulate the threat of incoming pain and reported lower subjective feelings of experienced pain^29,30^. On the other hand, when the sharing of pain is simultaneous and bidirectional, people may exhibit mutual amplification of pain experiences^12,13^ and physiological convergence in the low-to-high direction as found here. Investigating these differences in relation to social settings in future research would likely be illuminating.

Our study also indicated that individual-level traits influenced pair-level physiological similarity during face-to-face interaction. Specifically, lower AQ score in a pair predicted physiological similarity at the pair level. Research on Autism Spectrum Disorder (ASD) has shown that individuals with ASD tend to pay less attention to others’ expressions of distress^31^, and gaze less into another’s eyes^32,33^. Similarly, research on typically developed participants (without ASD diagnosis) showed that those with low AQ scores looked more at the other in real-time social interactions than those with high AQ scores^34^. Given these findings, a lower-AQ individual might attend more to another’s reactions than a higher-AQ individual. We thus conjecture that at least one person may have to attend to their counterpart’s facial reactions if physiological convergence is to be realized at the pair level.

In contrast, we found no significant relationship between trait empathy and physiological similarity. Previous research examining empathic responses to sufferers in pain has described a relationship between trait empathy and empathic reactions (e.g., the activity of the pain-related neural circuit)^2,6^. For example, Goldstein *et al*. showed that trait empathy modulated “physiological coupling” at the pair level when a participant observed and touched a partner who was in pain^30^. On the other hand, Martin *et al*.^13^, who used a similar bidirectional paradigm to that used in this study, showed no correlation between the sharing of subjective pain and empathic traits. This could mean that the absence of correlation is related to differences in interaction between the two social settings. As of yet, we can form no concrete conclusions about this point.

Our study has several limitations that should be addressed in future research. First, while our findings showed that the physiological responses of the pair converged asymmetrically over time, and that they also showed a greater similarity within each trial in a face-to-face interaction, we could not fully articulate the causal relationship between the two phenomena. To test whether inter-trial influence causes intra-trial similarity of physiological responses in a statistically rigorous manner, it will be necessary to increase the number of trials with the same intensity while minimizing participants’ habituation to the stimulus. Because there were only two trials for each level of stimulation in our study, we did not have sufficient statistical power for this analysis. Related to this point, we used only an ascending series of stimulation. It is thus not possible to conclude whether the saturation of the low-to-high convergence effect later in the stimulus sequence (Fig. 2a) was due to the higher stimulus intensities or simply to repeated interaction. In order to address this issue, random stimulation with different intensities in the sequence will be necessary. If the repeated interaction is important, saturation of the low-to-high convergence effect would persist even with random stimulation. On the other hand, if the stimulus intensity matters, the low-to-high convergence effect would be observed only with low intensity. Given that lower temperatures are more ambiguous in causing pain experiences, people may be influenced more by those who respond more strongly, as implied by the social learning strategy of “copy when uncertain”^35–37^.

Second, although our results indicated that a face-to-face setting is important for physiological convergence, the specific information that was used in the face-to-face condition (or unavailable in the shielded condition) remains unclear. Some bodily reactions to painful stimuli (e.g., reactions in the lower arm and hand on the side where the painful stimulus was administered) were also observable under the shielded condition (Fig. 1), so facial expression (e.g., eye movement, pupil size, blink, and/or facial muscular activity) or eye contact seem likely to be the main channels for physiological convergence. One possible process that can mediate physiological convergence is spontaneous mimicry. Pain is often expressed in facial reactions^38^, and observation of emotional faces activates relevant facial muscles^20,22,23^. Pupil dilation and blink reflex also reflect arousal change due to painful stimulus^39,40^, possibly causing spontaneous mimicry of pupil size^21^ among observers. It is noteworthy that the magnitudes of behavioural reactions (or any visual or audible responses during interaction) by the participants were so subtle that the experimenter did not notice them. Future research that combines the present paradigm with simultaneous measurements of participants’ attention (e.g., gaze direction), facial expressions (e.g., muscular activities, eye-blink, pupil size) and eye contact will be fruitful for specifying the critical channels of emotional contagion at the physiological level. In addition to such visual information, other modalities may also modulate physiological convergence in pain. For example, touching a partner, which is known to modulate physiological coupling between an observer and a sufferer^30^, appears to be an interesting possibility.

In summary, we have shown that bidirectional face-to-face interaction induces the convergence of emotional responses to pain at the physiological level. We have also observed asymmetric convergence in which the weaker responder increased their physiological responses to nearly match their partner’s responses over time. Since such an asymmetric process could have cumulative impacts on a group, it would be interesting to evaluate physiological contagion in a larger group interaction. The asymmetric convergence found in our study will help us to make empirically falsifiable predictions about larger group phenomena such as crowd joy^41^, panic, or mass hysteria^42^, in order to build a better understanding of collective dynamics.

## Methods

### Ethics statements

This study was approved by the Institutional Review Board of the Centre for Experimental Research in Social Sciences at Hokkaido University, in accordance with the Declaration of Helsinki. Written informed consent was obtained from all participants before beginning the experiment.

### Participants

Seventy-eight volunteers (38 females and 40 males; mean age: 20.5 ± 0.85 years) who were students at Hokkaido University participated in the experiment and received 2,000 yen (approximately 20 USD) as compensation for their participation. All the participants were recruited individually so that they had no interactions prior to the experiment. The participants were randomly assigned to either the face-to-face or the shielded condition. Each pair was sex-matched. Because the participants in each pair had not met prior to the experiment, we provided an opportunity for a short conversation at the beginning to reduce any social stress that might arise from interacting with an unfamiliar person.

When they were recruited, the participants were informed that in the experiment, they would be presented a series of thermal stimuli to their arm that would cause pain without damaging the skin. It was emphasized that they could refuse to receive further stimuli and leave the experiment at any time with full compensation. Through a series of pilot tests, we had confirmed that the thermal stimuli to be used in the study caused no physical damage to the skin. The participants who agreed to the procedure were asked to provide written informed consent. After the experiment, all the participants were encouraged to cool their arms with a cold towel. We confirmed that they had no physical damage or feeling of pain in their arms after the experiment.

### Conditions

Face-to-face condition: The two participants in each pair were seated face-to-face. They could see their partner’s upper body including the face, but were not allowed to communicate verbally.

Shielded condition: The two participants in each pair were seated as in the face-to-face condition, but they were prevented from viewing each other’s upper bodies including the face by a small partition placed between them (see Fig. 1).

### Stimuli

This experiment was carried out by two experimenters – one for each participant in the pair. Two aluminium rods were heated in water using a spirit lamp (Fig. 1). Once the water temperature had reached the target level, one experimenter took both aluminium rods from the water and passed one rod to the other experimenter. Next, after a 5-second verbal countdown, the experimenters applied the heated rods to the right arms of both participants simultaneously. The verbal countdown was delivered by one experimenter, who referred to a visual countdown on a computer screen that only the experimenter could see. The experimenters were trained to keep the difference in their stimulation timing below 500 milliseconds. The rod was applied for about 300 milliseconds in each trial. To habituate the participants to the aluminium rod, the rod was warmed in 40 °C water and applied to their right arms before the beginning of the task. The thermal stimuli, which increased from moderate to higher temperatures in 5-degree steps, were sequentially applied to the participant’s right arm a total of 12 times, with each stimulus temperature applied twice before moving to the next level (i.e., 40 °C, 40 °C, 45 °C, 45 °C, 50 °C, 50 °C, 55 °C, 55 °C, 60 °C, 60 °C, 65 °C, 65 °C). The stimulus durations and magnitudes were determined by reference to a previous study that evaluated sensitivity to thermal stimuli^43^. We also confirmed that this protocol caused no physical damage to participants’ skin by performing a series of pilot tests. In each trial, the experimenter kept the water temperature within plus or minus 1 degree of the target temperature. Although the participants were not aware of the precise temperature of the stimuli (they could not see the value on the thermometer), they were able to guess that it increased across the trials because they could see that the water was being kept warm with a spirit lamp. The stimuli and procedure for the shielded condition were identical to those used for the face-to-face condition, except for the small partition between the participants.

### Individual traits

In the post-session questionnaire, we administered the Japanese versions of the autism-spectrum quotient (AQ^25)^ and the trait empathic tendency sub-scales Perspective Taking (PT), Empathic Concern (EC), Fantasy (FS), and Personal Distress (PD) from the Interpersonal Reactivity Index (IRI^26)^.

### Procedure

The participants were randomly assigned to either the face-to-face or shielded condition. In each session, two participants were seated facing each other at a table. To reduce the potential stress of engaging in social interaction with an unfamiliar person, they were given the opportunity to have a two-minute conversation about a topic suggested by the experimenter (e.g., “What did you do last weekend?”). The experimenter then explained the process that would be used to record the participants’ physiological responses during the experiment. For each participant, a photoplethysmographic BVP device was placed on the tip of the left-hand middle finger. In the shielded condition, the experimenter placed a small partition on the table to prevent the participants from viewing other’s facial expressions. In each trial, two aluminium rods were heated until the water reached the target temperature. The experimenter then counted down from five to zero (“5, 4, 3,…”), and the heated rods were applied to the right arms of the participants simultaneously. In both conditions, the participants were instructed to direct their gazes forward during the entire task (though those in the face-to-face condition did not have to maintain eye contact with their partner).

### Index of physiological response to thermal pain

**Figure 4:**
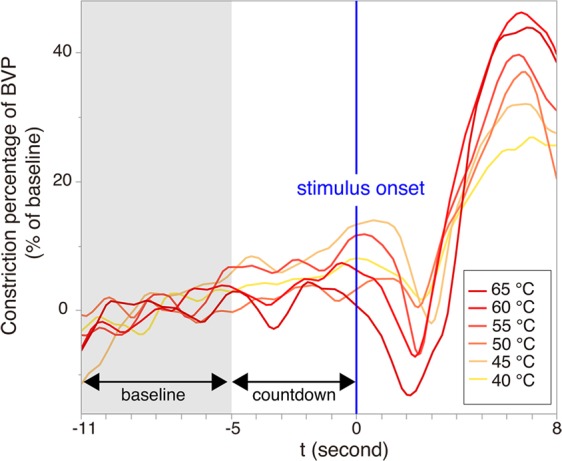
Example of BVP responses to thermal stimuli. An example time series of the mean constriction percentages of BVP in the face-to-face condition. For each thermal stimulus, the mean value for 6-second BVP amplitudes before the countdown was used as a baseline. Then, constriction percentage of the BVP amplitude from the baseline [ = 100 * (1 − signal-averaged BVP amplitudes after stimulus onset/baseline)] was calculated for each time point. For our analysis, we used the maximum of the 800 constriction percentage values after stimulus onset as a BVP response for each participant and each stimulus.

We recorded the photoplethysmographic BVP from each participant’s fingertip using a BIOPAC system (TSD200/PPG100C) to assess physiological arousal. For pre-processing, we down-sampled the BVP data from 2000 Hz to 100 Hz to reduce the calculation cost. We then evaluated the BVP amplitude by subtracting the negative envelope from the positive envelope. BVP amplitudes during 19-second intervals (from 6 seconds before the 5-second countdown to 8 seconds after the stimulus onset) were used in the analysis. Figure 4 shows how the BVP response was calculated over time from the BVP amplitudes. For our analysis of physiological influence, we calculated constriction percentage in the BVP amplitude from the baseline (the mean value of BVP amplitude in the 6-second interval before the countdown) after signal averaging to reduce the noise in the measurement for each of the 6 stimuli (i.e., 40 °C, 45 °C, 50 °C, 55 °C, 60 °C, 65 °C). The maximum constriction percentage after stimulus onset was used as the BVP response for each participant and each stimulus. For our analysis of the physiological similarity in each of the 12 trials, we used the BVP amplitudes at all 1900 time points during the 19 second interval.

### Data treatment and analysis

Equipment failure: BVP data from 3 pairs were excluded due to equipment failure, leaving us a total of 36 pairs (face-to-face condition: 18 pairs, shielded condition: 18 pairs) for analysis.

Missing data for individual traits: Several pairs did not provide responses for the IRI or autism-spectrum quotient (AQ) due to time limitations. Due to the order of the items in the post-session questionnaire (IRI before AQ), the number of missing data differed for the IRI (face-to-face condition: 2 pairs) and AQ (face-to-face condition: 5 pairs, shielded condition: 3 pairs).

### Linear mixed effects model (LMM) analyses for physiological influence

We conducted linear mixed effects model (LMM) analyses with reference to a standardized method for evaluating inter-trial physiological influence in dyads^44^. In the LMM analyses, the weaker and stronger responders were defined for each stimulus (40 °C, 45 °C, 50 °C, 55 °C, 60 °C) within each participant pair, based on their relative responses to the previous stimulation. The LMM analyses were performed on the responses to the remaining 6 stimuli. To centre the variables, the stimulus factors were converted into the differences from the first stimulus (45 °C: 5, 50 °C: 10, 55 °C: 15, 60 °C: 20, 65 °C: 25), and the BVP responses of the partner to the previous stimulus were centred so that the grand mean value was zero. The effect of the partner’s response to the previous stimulus (e.g., whether participant’s response to 50 °C was influenced by the partner’s response to 45 °C) was tested using an LMM. In the LMM, condition (the face-to-face or shielded condition), participant’s relative position (the weaker or the stronger responder within a pair), stimulus (45 °C, 50 °C, 55 °C, 60 °C, 65 °C), the partner’s BVP response to the previous stimulus, and their interactions were entered as fixed effects. Pair and participant were entered as random effects.

### Index of the physiological similarity of a pair

To evaluate the physiological similarity of each pair, cross-correlations were computed from the participants’ BVP data during the 19-second period of interest (12 times per pair). The response sequences were normalized such that the auto-correlation function at zero-lag was 1 by using the Matlab XCOV function with the ‘coeff’ option (MathWorks, Inc., Natick, MA). The resulting cross-correlation values varied between 1 and −1, representing a perfect positive and a perfect negative correlation, respectively. Since spontaneous emotional contagion is known to be evoked within 1000 ms of observing an emotional stimulus^20–23^, we adopted peak values for the correlation coefficients of a −1000 millisecond lag to a + 1000 millisecond lag as an index of the physiological similarity of each pair in each trial. To assess the similarity due to interaction, beyond the similarity resulting from the common thermal stimulus per se, we compared the similarities of the physiological responses of actual pairs with those of nominal pairs composed of two participants from different sessions (who experienced the same stimuli but were not paired during the task). Because the correlations of nominal pairs reflect only the similarity of the physiological responses induced by the same stimulation, a comparison of an actual pair’s similarity and a nominal pair’s similarity allows an evaluation of the extra similarity induced by interaction.

### LMM analyses of the effect of individual traits on physiological similarity

In the LMM analyses, the minimum or maximum score of individual traits (AQ score or trait empathy in IRI) in each pair and condition were entered as fixed effects. Pair was entered as a random effect. To centre the variables, the scores of individual traits were centred so that the grand mean value was zero.

## Supplementary information

Supplementary Information.

## Data Availability

The datasets used in the current study are available from the corresponding author on reasonable request.
