# Supplementary material for: Convergence of physiological responses to pain during face-to-face interaction
Source: Sci Rep. 2020 Jan 16;10:450. doi: 10.1038/s41598-019-57375-x (PMC6965106; doi:10.1038/s41598-019-57375-x)
Supplement: Supplementary file 1 — Supplementary Information. [file 41598_2019_57375_MOESM1_ESM.pdf]

Supplementary Information

Convergence of physiological responses to pain during face-to-face interaction

Aiko Murata[1,2], Hiroshi Nishida[3], Katsumi Watanabe[4,5], Tatsuya Kameda[6,7,8]*

1. NTT Communication Science Laboratories, Japan
2. Department of Behavioral Science, Hokkaido University, Japan
3. Faculty of Arts and Science, Kyushu University, Japan
4. Faculty of Science and Engineering, Waseda University, Japan
5. Faculty of Art & Design, University of New South Wales, Sydney, Australia
6. Faculty of Letters, the University of Tokyo, Japan
7. Center for Experimental Research in Social Sciences, Hokkaido University, Japan
8. Brain Science Institute, Tamagawa University, Japan

* Address correspondence to: tkameda@l.u-tokyo.ac.jp

Table S1. Fixed effects in full model for intertemporal physiological influence

Factor	*Est.(b)*	*CI*	*SE*	*t*	*p*
**(Intercept)**	**54.211**	**41.561 to 66.952**	**6.529**	**8.303**	**<0.001**
Stimulus	-0.030	-0.755 to 0.698	0.377	-0.081	0.936
Condition (face-to-face condition minus shielded condition)	-15.747	-32.931 to 1.405	8.821	-1.785	0.075
**Participant's relative position (weaker responder minus stronger responder)**	**-24.728**	**-42.076 to -7.404**	**8.961**	**-2.760**	**0.006**
Partner's response in the previous stimulus	0.025	-0.435 to 0.488	0.239	0.103	0.918
**Stimulus: Condition**	**1.048**	**0.093 to 1.994**	**0.488**	**2.150**	**0.032**
Stimulus: Participant's relative position	0.835	-0.247 to 1.906	0.555	1.504	0.134
Condition: Participant's relative position	21.643	-1.418 to 44.52	11.882	1.822	0.069
Stimulus: Partner's response	-0.004	-0.034 to 0.025	0.015	-0.289	0.773
Condition: Partner's response	-0.274	-0.928 to 0.385	0.338	-0.812	0.417
Participant's relative position: Partner's response	-0.230	-1.081 to 0.619	0.430	-0.535	0.593
Stimulus: Condition: Participant's relative position	-0.903	-2.334 to 0.532	0.742	-1.218	0.224
Stimulus: Condition: Partner's response	0.017	-0.023 to 0.057	0.021	0.806	0.421
Stimulus: Participant's relative position: Partner's response	-0.005	-0.056 to 0.047	0.026	-0.188	0.851
**Condition: Participant's relative position: Partner's response**	**1.208**	**0.095 to 2.323**	**0.567**	**2.132**	**0.034**
Stimulus: Condition: Participant's relative position: Partner's response	-0.057	-0.124 to 0.01	0.035	-1.647	0.100

Note. CI indicates 95% confidence interval of the fixed effect.

Table S2. Fixed effects in models for physiological influence in the face-to-face condition.

Factor	*Est.(b)*	*CI*	*SE*	*t*	*p*
**(Intercept)**	**36.961**	**24.884 to 48.952**	**6.167**	**5.993**	**<0.001**
**Stimulus**	**1.060**	**0.446 to 1.668**	**0.307**	**3.456**	**<0.001**
Participant's relative position in the previous stimulation (weaker responder minus stronger responder)	-0.104	-14.674 to 14.227	7.440	-0.014	0.989
Partner's response in the previous stimulus	-0.287	-0.735 to 0.172	0.228	-1.258	0.210
Stimulus: Participant's relative position	-0.140	-1.067 to 0.783	0.462	-0.304	0.762
Stimulus: Partner's response	0.012	-0.014 to 0.039	0.014	0.900	0.370
**Participant's relative position: Partner's response**	**0.976**	**0.292 to 1.657**	**0.353**	**2.765**	**0.006**
**Stimulus: Participant's relative position: Partner's response**	**-0.063**	**-0.105 to -0.022**	**0.021**	**-2.966**	**0.004**

Note. CI indicates 95% confidence interval of the fixed effect.

Table S3. Fixed effects in models for physiological influence in the shielded condition.

Factor	*Est.(b)*	*CI*	*SE*	*t*	*p*
**(Intercept)**	**54.953**	**42.552 to 67.521**	**6.274**	**8.758**	**<0.001**
Stimulus	-0.001	-0.713 to 0.719	0.370	-0.003	0.998
**Participant's relative position in the previous stimulation (weaker responder minus stronger responder)**	**-24.851**	**-43.169 to -6.622**	**9.418**	**-2.639**	**0.009**
Partner's response in the previous stimulus	0.047	-0.43 to 0.532	0.248	0.191	0.849
Stimulus: Participant's relative position	0.748	-0.422 to 1.886	0.584	1.281	0.202
Stimulus: Partner's response	-0.004	-0.035 to 0.027	0.016	-0.252	0.802
Participant's relative position: Partner's response	-0.294	-1.194 to 0.616	0.442	-0.665	0.507
Stimulus: Participant's relative position: Partner's response	-0.001	-0.055 to 0.053	0.027	-0.054	0.957

Note. CI indicates 95% confidence interval of the fixed effect.

Table S4. Fixed effects in models for the effect of AQ on physiological similarity

Model	*Factor*	*Est.(b)*	*CI*	*SE*	*t*	*p*
Model with the minimum AQ in pair	**(Intercept)**	**0.296**	**0.212 to 0.38**	**0.045**	**6.644**	**<0.001**
	Condition (face-to-face condition minus shielded condition)	-0.020	-0.143 to 0.103	0.065	-0.303	0.765
	Minimum AQ in pair	0.005	-0.007 to 0.017	0.006	0.793	0.436
	**Condition: Minimum AQ in pair**	**-0.027**	**-0.048 to -0.007**	**0.011**	**-2.471**	**0.021**
Model with the maximum AQ in pair	**(Intercept)**	**0.262**	**0.181 to 0.343**	**0.043**	**6.083**	**<0.001**
	Condition (face-to-face condition minus shielded condition)	0.131	0 to 0.262	0.070	1.885	0.072
	Maximum AQ in pair	0.010	-0.007 to 0.027	0.009	1.146	0.263
	Condition: Maximum AQ in pair	-0.023	-0.045 to -0.002	0.012	-2.015	0.055

Note. CI indicates 95% confidence interval of the fixed effect.

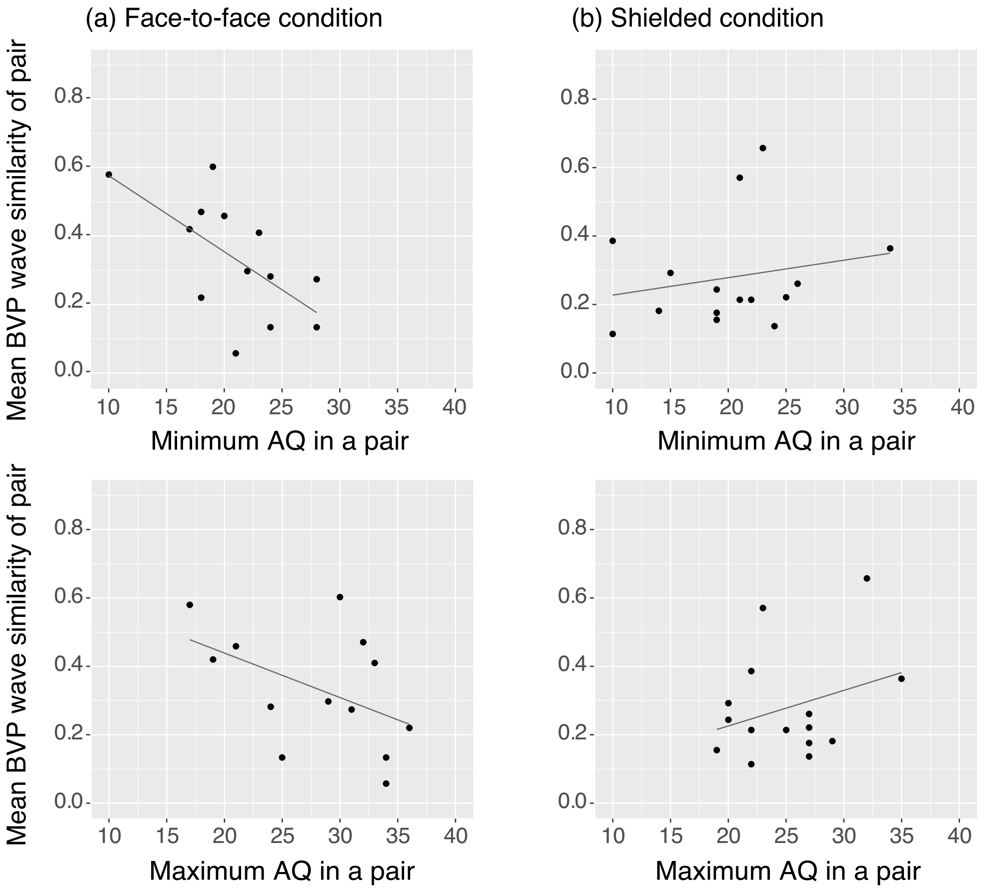

Figure S1. Scatterplots showing the relationship between AQ and mean BVP wave similarity within pairs in (a) the face-to-face condition and (b) the shielded condition.

Mean BVP wave similarity was calculated by averaging the BVP wave similarity values of all trials for each pair.
